# Formation of Gold Microparticles by Ablation with Surface Plasmons

**DOI:** 10.3390/nano3040592

**Published:** 2013-10-28

**Authors:** Quincy Garner, Pal Molian

**Affiliations:** Laboratory for Lasers, MEMS and Nanotechnology, Department of Mechanical Engineering, Iowa State University, Ames, IA 50011, USA; E-Mail: molian@iastate.edu

**Keywords:** laser, plasmons, gold, nanoparticles, porous alumina membrane

## Abstract

The formation of gold microparticles on a silicon substrate through the use of energetic surface plasmons is reported. A laser-assisted plasmonics system was assembled and tested to synthesize gold particles from gold thin film by electrical field enhancement mechanism. A mask containing an array of 200 nm diameter holes with a periodicity of 400 nm was prepared and placed on a silicon substrate. The mask was composed of 60 µm thick porous alumina membrane sputter-coated with 100 nm thin gold film. A Nd:YAG laser with 1064 nm wavelength and 230 µs pulse width (free-running mode) was then passed through the mask at an energy fluence of 0.35 J/cm^2^. The extraordinary transmission of laser light through alumina/gold micro-hole optical antenna created both extended and localized surface plasmons that caused the gold film at the bottom of the mask to fragment into microparticles and deposit on the silicon substrate that is in direct contact with the mask. The surface plasmon method is simpler, quicker, more energy efficient, and environmentally safer than existing physical and chemical methods, as well as being contamination-free, and can be extended to all types of materials that will in turn allow for new possibilities in the formation of structured surfaces.

## 1. Introduction

Plasmonics is a rapidly emerging technology for photonics, sensors, microscopy, data storage, and lithography. However, there is little work reported on the use of plasmonics for nanoscale manufacturing applications such as nanomachining and nanoparticle synthesis [1,2,3]. The coherent interference of surface plasmon polaritons was able to produce 50–70 nm diameter holes in silicon wafers [1]. Plasmonic effects strongly affected the aspect ratio of nanometer-sized holes in femtosecond laser ablation if the surface plasmon resonance conditions were met [2]. Nanofragmentation of gold thin films by 532 nm, 10 ns laser radiation in the system air/gold film/glass was achieved at surface plasmon resonance conditions [3]. The plasmonic effects aided in reducing the energy fluence required for thermal ablation and permitted self-organization of micro-ablation events [3].

Gold nanoparticles play a vital role in biology, chemistry, optics and microelectronics by virtue of a large surface-to-volume ratio, quantum confinement, and other unique properties. For example, gold nanoparticles are extensively used in life sciences for labeling, delivery, heating, and sensing due to their strong absorption, scattering, plasmon resonance, X-ray contrasting and functionalization with ligands. The development of highly ordered, uniform gold nanoparticles is also useful to the advancement of plasmonic devices. Gold nanoparticles could act as a surface coupler and, under certain size and periodicity conditions, can help in the transmission of sub-diffraction limited light. New applications of gold nanoparticles are constantly emerging in many areas such as fuel cells, cancer cell therapy, nanoimprinting and solar cells [4,5,6].

Many physical and chemical techniques are available to generate gold nanoparticles [7,8,9,10,11,12,13,14,15,16,17,18,19,20]. Pulsed laser ablation (PLA) in gas and liquid media appear to lead among all physical processes. When the energy fluence of a laser beam exceeds that of the ablation threshold of gold, fragmentation occurs in the form of ions, electrons and neutrals at the irradiated spot forming nanoparticles. Despite the availability of a number of physical and chemical processes for gold nanoparticles, there are still many issues that need to be addressed in the form of particle size and uniformity, production rate, simplicity, contamination, energy efficiency and system cost. In this paper, we present a method capable of producing gold nanoparticles through the excitation of surface plasmons that addresses many of these problems.

## 2. Theory

Plasmonics has been known by scientists for many decades, yet its applications to photonics, microelectronics and medicine have just begun to be realized within the past decade [21]. Surface plasmons (SP) are the collective and coherent oscillations of conduction electrons in a metal film present at the interface of metal-dielectric with permittivities of opposite sign. Energy can be harnessed when the electrons are coupled with photons by creating quasiparticles called polaritons. SPs are easier to produce in metals that exhibit a dielectric function with large negative real number and small imaginary number. Common metals that fulfill this requirement under certain wavelengths are silver, gold and aluminum.

**Figure 1:**
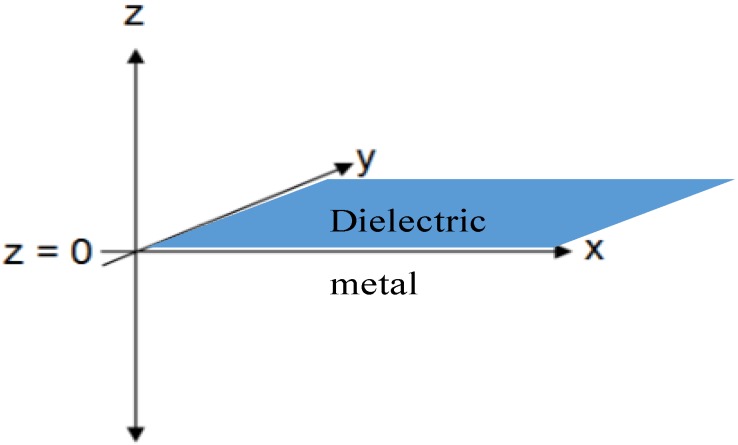
The coordinate system used for the description of surface plasmons.

SPs propagate along the interface between a metal and dielectric, which is typically perpendicular to the incident light. Figure 1 shows a coordinate system where *z* > 0 is the dielectric, *z* < 0 is the metal, and the metal-dielectric interface is in the X-Y plane.

It may be noted that SPs are purely longitudinal surface charge density waves unlike other electromagnetic waves. The plasmon wavevector, *k_x_*, is related to the angular frequency, ω, of the incident light through the dispersion relationship [22]:

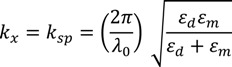
(1)
where *k_x_* = *k_sp_* = 2π/λ*_sp_* with λ*_sp_* the plasmon wavelength, λ_0_ the incident laser wavelength, ε*_m_* (λ_0_) and ε*_d_* (λ_0_) are the dielectric functions of the metal and dielectric material, respectively. An important attribute of SPs is their potential for a shorter wavelength and higher intensity compared to the incident light. It should be noted that the dielectric function of metal is a complex wavevector with ε*_m_* = ε*_m_*ʹ + *i*ε*_m_*ʺ while for the dielectric it is a real number. The amplitude of SPs decreases with increasing propagation distance in the X-direction and eventually dissipates. The propagation distance *L* along the metal surface is given by:

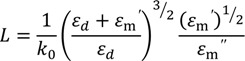
(2)


The wavevector perpendicular to the interface, *k_z_*, corresponds to electric fields that decay exponentially with increasing distance from the interface. The penetration depth is defined as the distance from the interface of metal-dielectric at which the amplitude is reduced to 35% of the initial value. The penetration depths and permittivites are related by:

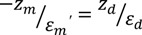
(3)
where *z_m_* and *z_d_* are the penetrations in metal and dielectric respectively. The penetration depth in metal is usually smaller than in dielectric due to considerable energy losses.

The extraordinary optical transmission phenomenon, originally discovered in nano-hole arrays of metal thin films, is attributed to the excitation of SPs that exist in two basic forms: localized (LSP) and extended (ESP) [23]. LSPs are localized electromagnetic fields near the surface of isolated nanostructures while ESPs propagate in the horizontal plane. LSP depends on the shape and size of the hole while the ESP is a function of the periodicity. Both SPs contribute to the enhanced transmission. In most cases, SPs are not easily excited due to the momentum difference between the incident light waves and the waves of SPs. To compensate for this, periodic arrays in thin films can be properly tuned to account for the momentum mismatch and consequent excitation of SPs [24,25].

The transmission of light in a single sub-wavelength aperture can be enhanced due to the existence of LSPs. For periodic sub-wavelength apertures, the total transmission is based on the integrated effect of LSP and ESP. The maximum transmission depends on the period of the nano-hole array, the incidence angle and the polarization of the excitation light. The transmission of light in nano-hole arrays is much higher than expected from classic diffraction theory implying that even the light impinging on the metal between the holes can also be transmitted. In other words, the whole periodic structure acts like an antenna in the optical regime. The transmission spectra of hole array display peaks that can be tuned by adjusting the period and the symmetry is given by the following equation:

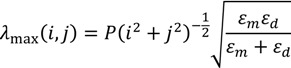
(4)
where λ_max_ (*i*,*j*) is the wavelength of peak light transmission, *P* is the periodicity, *i* and *j* are the scattering orders from the array, ε*_m_* and ε*_d_* are respectively the dielectric constant of the metal and the dielectric material. Each peak of the light transmission is labeled by a set of integers (*i*,*j*).

The oscillatory nature of the surface modes enables the resonant enhancement of the highly confined electromagnetic fields. At the resonance, the intensity of the electric field at the interface between the metal and the dielectric is strongly enhanced due mainly to the smaller complex permittivity of the dielectric compared to the metal. SPs can enhance the electrical field by as high of a factor as 1000. Nanofocusing, or the strong localization of the optical energy in regions smaller than possible by the diffraction limit, can offer promising applications in nanofabrication. In summary, when the incident light illuminates the nano-hole array, localized and extended SPs are excited under surface plasmon resonance conditions. The nano-hole array with SPs as electrical dipoles acts as an optical antenna with the potential capability for nanoscale material removal.

## 3. Experiments

**Figure 2:**
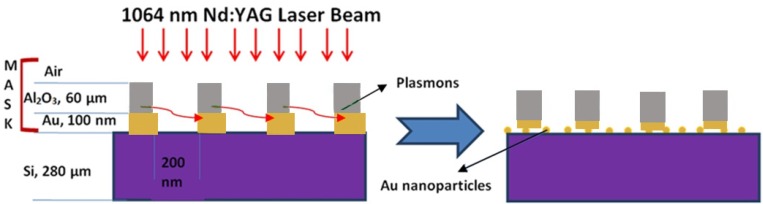
Schematic diagram of experimental excitation of surface plasmons and the resulting formation of microparticles.

Figure 2 illustrates the underlying physical mechanism involved in the experiment and its projected effect on the formation of metallic microparticles. In this setup, there are three main components: metal/dielectric mask, silicon substrate and 1064 nm laser.

**Figure 3:**
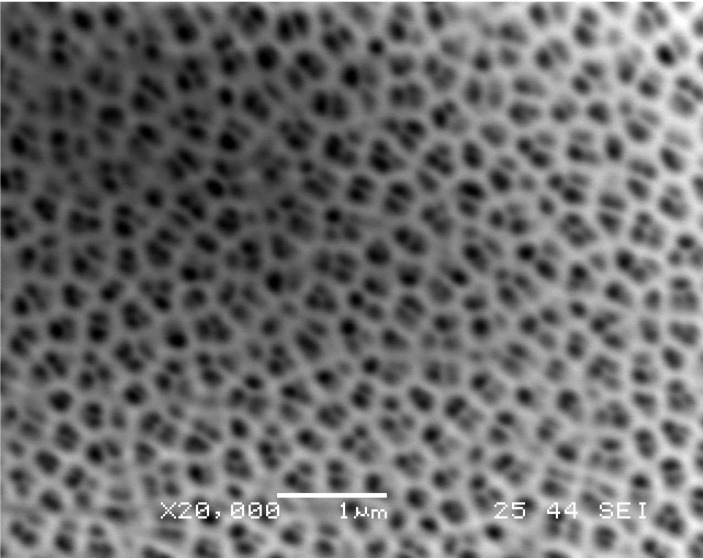
SEM image of porous alumina membrane.

The mask is prepared as follows: porous alumina membranes with a diameter of 25 mm (Anodisc25™, GE Healthcare, Piscataway, NJ, USA) were received from Whatman. Anodisc25 is composed of a high purity alumina matrix manufactured by electrochemical methods. It has a precise, non-deformable honeycomb pore structure with no lateral crossovers between individual pores. The membrane has an average thickness of 60 µm, pore diameter of 200 nm and periodicity of 400 nm. Figure 3 shows the scanning electron micrograph image of the membrane. Sputtering was then employed using a Denton Vacuum Desk V (Denton Vacuum, Moorestown, NJ, USA) at 40 m Torr of pressure for 200 s to deposit a 100 nm thin film of gold on the bottom side of the membrane. The dielectric alumina micro-hole array pattern and thin metal gold film combination serves as an optical antenna for light transmission.

Silicon wafers [p-type Si:B (100)] were acquired from University Wafers, MA. The wafers had a diameter of 25 mm and thickness of 280 ± 25 µm. One side of the wafer was polished while the other side was etched using an alkaline solution.

The laser system used was a Q-switched Nd:YAG laser (INDI series, Spectra-Physics, Santa Clara, CA, USA) along with beam delivery system and positioning table. The laser can be operated in Q-switched mode with a pulse width of 10 ns or in free-running mode with a pulse width of 230 µs. The maximum average power of the laser is 3 W with a maximum possible repetition rate of 10 Hz.

The experiments consisted of placing the gold-sputtered membrane in direct contact with the polished side of the silicon wafer in an ambient, low humidity environment followed by the passage of the Nd:YAG laser beam through the mask. The 6 mm diameter, p-polarized beam emitted from the laser resonator was delivered to the mask through a 90 degree steering optics without focusing by a lens. The pulse repetition rate was held constant at 1 Hz. Only a single pulse was used for each experiment. The average power of the laser was varied from 0.1 to 0.5 W. Two pulse widths—10 ns and 230 µs—were investigated. In addition, experiments were conducted with masks having either only top side gold thin film or without any gold film. Optical microscopy, scanning electron microscopy, and atomic force microscopy were then used to examine the particle size and distribution of gold particles. A histogram was made to relate the particle size distribution and its relationship with the array of micro-holes in the mask.

## 4. Results

Nine different experiments involving both short and long pulsed beams were carried out, with multiple trials performed for each setup. Table 1 lists the specific experimental parameters for each setup. The results of these nine experiments are described below.

**Table 1:** Experimental trials and parameters.

Setup Number	Pulse Width (FWHM)	Average Power	Peak Power	Energy Fluence	Mask Orientation (Alumina)
1	10 ns	0.45 W	45 MW	1.6 J/cm^2^	Gold on bottom
2	10 ns	0.34 W	34 MW	1.2 J/cm^2^	Gold on bottom
3	10 ns	0.23 W	23 MW	0.8 J/cm^2^	Gold on bottom
4	10 ns	0.10 W	10 MW	0.35 J/cm^2^	Gold on bottom
5	230 μs	0.10 W	435 W	0.35 J/cm^2^	Gold on bottom
6	230 μs	0.10 W	435 W	0.35 J/cm^2^	No gold coating
7	230 μs	0.23 W	10 kW	0.8 J/cm^2^	No gold coating
8	10 ns	0.23 W	23 MW	0.8 J/cm^2^	No gold coating
9	230 μs	0.10 W	435 W	0.35 J/cm^2^	Gold on top

**Figure 4:**
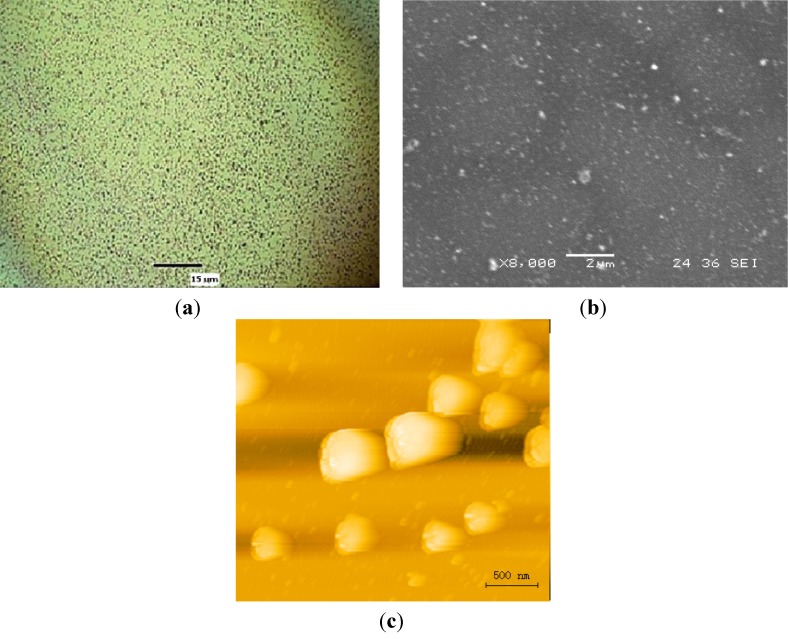
Gold nanoparticles on the surface of silicon substrate (setup 5). (**a**) Optical image, (**b**) SEM image and (**c**) AFM image.

**Figure 5:**
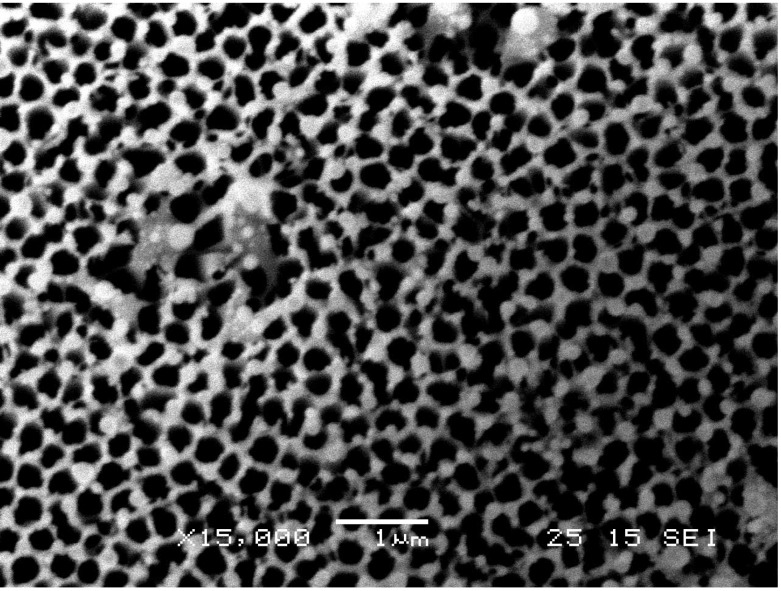
SEM image of the mask after laser irradiation.

In experimental setups 1–3, the alumina membrane was completely destroyed in the irradiated area due to the high intensity of SPs. The pore structure of the alumina in this area was damaged and only fragmented pieces of alumina and gold remained in the exposed area. In experimental setups 4 and 5, a noteworthy result occurred. In these trials, the laser beam irradiated the alumina membrane resulting in clean ablation and subsequent deposition of gold particles on the surface of the silicon substrate. The pore structure of the alumina membrane in this case was undamaged while the gold underneath the alumina in the exposed area was removed and deposited onto the wafer’s surface. Figure 4 shows the microparticles embedded on silicon. Figure 5 shows the SEM image of the mask after laser irradiation. The intriguing aspect of this experiment is the low energy fluence and long pulse width of the laser beam in setup 5 that was needed for the fragmentation of the gold thin film. Various studies of nanosecond pulsed laser ablation in gas or liquid media indicate that threshold fluence required for gold thin films is approximately 5–8 J/cm^2^ [3]. For the femtosecond pulsed laser ablation, threshold fluence is approximately 1.5 J/cm^2^ [26]. For long pulses such as microseconds, threshold fluence is expected to be much higher. In the described work, 0.35 J/cm^2^ was sufficient to ablate a significant amount of gold in the exposed area. A comparison with direct laser ablation suggests that surface plasmons substantially reduced the threshold fluence for ablation of gold. To further validate this result, setup 5 was repeated several times under the same conditions while irradiating different sections of the gold/alumina mask. Each of these new tests yielded the same result as before with the formation of gold microparticles on the surface of the substrate and no visible damage to the pore structure of the alumina membrane.

Under infrared wavelengths, gold has been shown to induce plasmons at the surface of a dielectric interface [27]. In this case, the alumina membrane acts as both the dielectric material and a periodic enhancer of localized surface plasmons. The 200 nm pores in the alumina membrane serve as the periodic sub-wavelength apertures in the system as discussed in the Theory section. Since the gold layer is deposited via electron beam deposition, the resulting layer takes on the shape of the supporting alumina layer, allowing for the resonant enhancement to occur on the semi-periodic gold layer. By inducing surface plasmons at the surface of the gold interface, microablation of the gold can occur at energy levels much lower than that of direct laser ablation. To lend credence to this observation, we will examine the results from experimental setups 6–9. In setup 8, an alumina membrane without a gold coating was subjected to the same orientation, peak power, and pulse time as setup 3.

In this setup, the alumina membrane pore structure was completely undamaged at the location of irradiation, suggesting that the alumina membrane was not damaged as a result of the initial energy of the laser in experimental setups 1–3, but as an effect of the creation of a surface plasmon wave at the interface of the gold/alumina. In setup 9, the gold-coated alumina membrane was placed in contact with the silicon substrate as before with the exception that the gold coated side was placed facing up and the non-coated side of the mask was in direct contact with the substrate. A long pulse with peak power of 0.1 W was used in a similar fashion as in setup 5. Following exposure by the Nd:YAG laser, the pore structure of the alumina membrane in setup 9 was found undamaged; however, no ablation or deposition of metallic gold nanoparticles had occurred. It is apparent that the alumina membrane is important in the production of surface plasmons in our setup due to its favorable dielectric properties and periodic nature. In many cases, air can act as the dielectric medium for inducing surface plasmons at the interface of a metal. In setup 9, the air/gold interface was unable to resonantly excite the strong surface plasmon wave that is needed to result in the ablation of gold.

**Figure 6:**
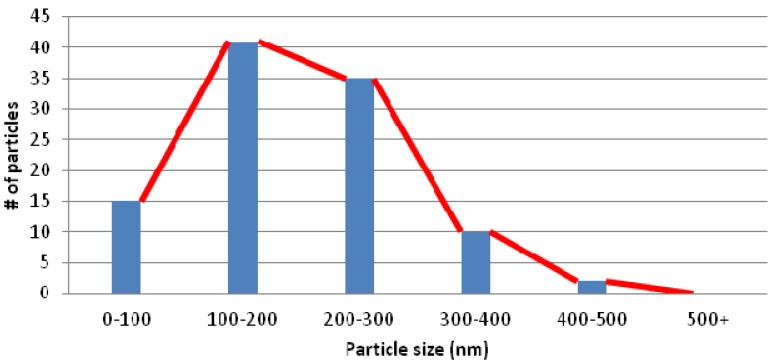
Size distribution of gold nanoparticles on the silicon substrate.

Now that we have examined the mechanism behind the formation of gold particles in this experiment, we can begin to discuss the size and distribution of these gold particles. Following the deposition, several optical and SEM images were taken of the gold particles on the surface of the silicon substrate. Upon analyzing these images, it is clear that the majority of the particles found on the surface have diameters in the range of 100–300 nm. Figure 6 shows a histogram of the average distribution of particle size per 15 μm square area. Results are consistent with the size of the pores in the alumina membrane and suggest that the size of the particles can very likely be easily influenced by adjusting the pore size of the alumina membrane. The controllability of the size of gold microparticles is a highly sought after trait due to its ever-growing applications in medicine and plasmonics based nanofocusing devices.

While the particles shown in the images presented do not appear to be highly periodic in nature, there does appear to be an even distribution of particles on the surface of the substrate. As evidenced by the images of the mask following exposure, it appears that the ablation of the gold generally occurs at the pores or near the edges of the pores in the mask and not the actual alumina. Because of this, the gold particles formed on the surface of the substrate take on the evenly distributed nature of the membrane and provide a thin monolayer of particles. Upon initial deposition, the gold nanoparticles in reality may have a slightly more periodic nature than what is shown in the optical and SEM images. After the experiment is performed, the surface of the substrate could potentially be disturbed due to the nature of moving the wafer during examination and imaging.

## 5. Discussion

The results of the experiment appear to show that highly energetic surface plasmons are formed during our process, resulting in the fragmentation of thin gold film to microscale particles. To validate this claim, we will examine whether surface plasmon resonance (SPR) conditions are met in our experiments. For 1064 nm wavelength light, the dielectric constant of alumina is a real number with a value of 10, while the dielectric constant of gold is a complex number with a value of −50 + 4*i* [28]. Application of Equations (1) and (4) in the present study provide that the wavelength of the SP is 300 nm and periodicity for SPR condition to occur is 300, 425 and 602 nm (first, second and third resonance modes), respectively. In our case, the wavelengths of the surface plasmons are much shorter than the incident light and the second resonance mode of the periodic enhancement matches the average period of the mask.

The most exciting result of the study is the ability for SPs to ablate gold thin film at low energy fluence for long pulse width of incident laser light. For a gold target directly ablated by a laser, threshold fluence is given by [29]:

Threshold (J/cm^2^) = 0.049 (pulse width in ps)^1/2^ (5)

Application of Equation (5) in direct laser ablation yields a threshold fluence of 743 J/cm^2 ^ for 230 µs pulse and 0.5 J/cm^2^ in 10 ns pulse. It may be noted that threshold fluence is also a function of wavelength which is not displayed in Equation (5). The fact that SPs require only 0.35 J/cm^2^ for the 230 µs incident light indicates that the mechanism of material removal in this process is different from typical thermal ablation encountered in 1064 nm laser irradiation. In a study on the 532 nm, 10 ns pulsed laser irradiation of the system air/gold film/glass under the conditions of surface plasmon resonance (SPR), a threshold fluence of 5.5 J/cm^2^ was reported to produce gold nanoparticles [3]. Without SPR conditions, threshold fluence was found to be 8 J/cm^2^. The mechanism accounting for material fragmentation under SPR conditions was thermal phase transition (melting and consequent micro-abalation) with a corresponding space modulation and subsequent partial nanostructure formation [3].

Results obtained in the present work did not offer any evidence for thermal damage like melting or evaporation thus eliminating thermal ablation as a possible mechanism. The non-thermal ablation mechanisms include Coulomb explosion and electrical field intensity evaporation. Typically an atom (ion) can be removed from a solid if its total energy exceeds the binding energy (*i.e.*, the energy of vaporization per particle). In a metal like gold, Coulomb explosion works under very high intensity and short pulse duration of the energy source because all energy losses due to electron-ion Coulomb collisions and heat conduction must be negligible. While the intensity of the laser light used during the experiment would not normally result in Coulomb explosion in gold film, the added effect of the extraordinary surface plasmon wave and adiabatic focusing allows for significantly higher effective intensities to be produced and for Coulomb explosion to occur in the gold ions. The authors believe the operating mechanism in the present study is electrical field enhancement in the vicinity of micro-holes where field-induced repulsive forces caused thin film fragmentation. The metal/dielectric mask works like an optical antenna exciting a huge electric field enhancement at the interface between alumina and gold film. Modeling the micro-hole array mask as a dipole antenna, the increase in electrical field intensity is dependent on the shape, radius and length of micro-holes in the mask. Electric field intensity enhancement can be estimated by assuming a small taper in the hole in the porous alumina membrane and using Gramotnev’s model of adiabatic nanofocusing [30]. Different structures including sharp metal tips, dielectric conical tips covered in metal film, sharp V grooves and nanowedges, *etc.* have been suggested for nanofocusing of plasmons [30]. Here, we assume that the taper begins at one side of the pore in alumina and ends on the opposite opening at an infinitely sharp point. Due to the nature of the sputtering process, the top of the walls of the alumina pores will likely contain a thin layer of gold film, allowing us to approximate our system as a sharp V dielectric groove covered in metal film. From the pore diameter and thickness of alumina membrane, we approximate the taper angle β ≈ 0.0033. According to Gramotnev’s model of adiabatic nanofocusing [30], electrical field enhancement would occur if:

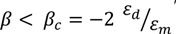
(6)
where ε*_d_* = 10, *ε_m_ʹ* = −50 and thus β*_c_* = 0.4. Since ˂β*_c_*, significant enhancement of the electric field can occur *assuming no plasmon energy dissipation in the metal*. Although the electric field will theoretically be infinite at the location of “zero radius” tip, this would not be the case in practice. At distances of 10^−2^ μm from the tip, the electric field enhancement is estimated to be around 100–150 times the normal value [30]. Thus, the high field enhancement factor induces dipole moments in gold and thereby pulling the atoms and grains out of gold.

The surface plasmon method described in this work is simpler, quicker, more energy efficient, and environmentally safer than existing physical and chemical methods, as well as being contaminant free. The technique can be readily extended to all types of materials. A well-known physical technique for gold is pulsed laser ablation (PLA) in vacuum or gaseous environment and is widely used to produce nanoparticles collected in the form of nanopowder [16]. Although PLA does not require high temperature or a chemical reaction, it is limited by the need for a high vacuum, high energy fluence, and long pumping time. In addition, the broader distribution of nanoparticles is a problem. An improved PLA for gold nanoparticles is performed by immersing the gold target in a liquid medium leading to functionalized gold nanoparticles with a ligand of choice in a colloidal solution. For example, 532 nm, 7 ns Nd:YAG laser ablation of gold target at 79 J/cm^2^ in distilled water produced colloidal gold nanoparticles [9]. The main difference between ablation in gas and in liquid is that liquid produces a stronger confinement of the expanding plasma plume generating higher temperatures and pressures and causing the vaporization of the liquid/chemical reactions and much broader distribution of nanoparticles [16,17,18,19]. It has been shown that laser ablation in liquid produces surface-charged nanoparticles with a shell of dipole molecules (e.g., water) formed around them, preventing agglomeration. Stable gold nanoparticles were synthesized by laser ablating gold foil placed inside ionic liquids without the addition of any external chemical reagent [18]. Commercialization of laser ablation in liquids was launched by *Particular GmbH* for the production of gold nanoparticles in a variety of biophotonic applications.

The other well defined process group used in the formation of gold particles is through chemical means, which is based on the synthesis of compounds to extract gold particles from a chemical system. Simple chemical reduction methods can produce 5–100 nm nanoparticles but the surface of these nanoparticles are often contaminated with reaction by-products such as anions and reducing agents, which can interfere with subsequent stabilization and functionalization steps [20]. Nakamoto [10] produced 11–76 nm gold particles by the controlled thermolysis of ammonium gold (I) thiolate. Arshi [11] used a hybrid method involving chemical mixing and microwave heating to produce average particle size of 4 nm.

While these are just a few example techniques used to manufacture gold nanoparticles, the applications of these techniques are just as diverse and far-reaching. Cherukuri and Curley [12] discussed the applications of gold nanoparticles in the treatment of malignant cells. Gold nanoparticles conjugated with cetuximab are shown to be quickly internalized by pancreatic and colorectal cancer cells in the human body. Following internalization, a non-invasive/non-ionizing radiofrequency field is focused in the affected area of the body. This exposure resulted in the heating of the gold nanoparticles and surrounding malignant cells. The treated cells showed a cytotoxicity rate of almost 100%. In the microelectronics industry, gold nanoparticles bring exciting new possibilities to photovoltaic cells and conventional lithography. Colloidal silver and gold nanoparticles are used to trap light on the surface of silicon photovoltaic cells [13]. By taking advantage of the plasmonic tendencies of gold nanoparticles, it has been shown that an enhancement of the photovoltaic conversion efficiency can occur. When compared to similar silicon solar cells without gold nanoparticles, the new solar cells showed a significant increase in the external quantum efficiency under visible and near-infrared light due to the effect of plasmonic light scattering. In lithography, the use of gold particles can be used to help effectively overcome the diffraction limit of light. Gold triangular nanoprisms patterened in a hexagonal lattice have been studied to observe their super focusing properties [14].

## 6. Conclusions

The work presented in this paper attests to the power of surface plasmons for the ablation of gold. The excitation of surface plasmon resonance described in this technique is a low energy alternative to the traditional pulsed laser ablation for the formation of structured particles on the surface of a substrate. The plasmonic system takes advantage of a novel use of porous alumina membrane as an effective dielectric with periodic sub-wavelength aperture enhancement of localized surface plasmons at the interface of a gold film. There is an orderly distribution of particles on the substrate with a close match between the particle size and the hole size in the mask. The results provide a strong basis for further development and applications related to laser-assisted surface plasmon excitation. Unlike other chemical techniques used to create metallic nanoparticles, the advancement of this process will allow for the deposition of particles over a broad area and allow for the particles to be transferred to any substrate in an evenly distributed fashion. The proposed technique is also advantageous over other laser assisted techniques in that the process requires a lower energy density than any other known technique. The applications of the proposed technique are far-reaching and could potentially impact the advancement of microelectronic and medicinal research.
